# Rupture of the Liver

**Published:** 1897-10

**Authors:** W. J. Martin

**Affiliations:** Kankakee, Ill.


### REPORTS OF CASES.

### RUPTURE OF THE LIVER.

By W. J. Martin, V.S.,
KANKAKEE, ILL.

On August 27th of this year I was called to see a black gelding, aged six years, weighing 1500 pounds, used for trucking purposes by a wholesale grocery firm of this city. The horse was first noticed to be sick at 4 o'clock, and I saw him half an hour later. At noon he ate his usual feed, and was, to all appearances, in perfect health. At the time of my examination he was still hitched to the truck with his mate.

He presented the following symptoms: Pulse quick and weak (70 per minute); mucous membrane of a pale, blanched color; ears cold and hanging pendulous; head hanging down in a listless manner; lower lips pendulous; legs and body very cold; temperature in rectum 101½° F.; respiration slightly above normal. Frequent attacks of dyspnœa, with reeling gait, when the animal was made to move backward or his head was raised higher than the natural position. Not the slightest abdominal pain was shown by the animal, nor was there any perspiration on any part of the body. When placed in his stall the horse would eat at times a small amount of hay between the attacks of dyspnœa, which would come on every few minutes. Diagnosis: Internal hemorrhage from some large organ. Prognosis: Death. This took place some time during the night.

Post-mortem August 28th, 10 A.M.: Body well nourished—in fact, very fat. On removing the skin from the body the subcutaneous tissues were seen to be stained a deep yellow color. This staining was most apparent over the loins and hind limbs. On opening the abdominal cavity a rush of blood took place in such an enormous quantity that it appeared as if the horse had been bled to death into his own abdomen. This was just what had happened. On the lower border of both the right and left lobes of the liver were large rents, extending through the outer serous covering, Glisson's capsule, and nearly through the parenchyma of the gland. The entire portion of the liver above the rupture was of a dark red color, smooth to the touch, and very dense in structure. The external covering of the gland—Glisson's capsule—was of a tough, leathery consistence. On section of the liver, numerous white spots were visible around the margin of the hepatic lobules, presenting that well-known appearance referred to as "nutmeg" liver. The parenchymatous structure of the liver was soft and easily broken down on pressure by the fingers. The portal vein was filled with a gelatinous plug of serum. The other bloodvessels of the gland were partly filled with coagulated blood of a dark brown color. No cicatrices of previous rupture of small bloodvessels nor of perihepatitis of the organ were to be seen upon the external surface of the liver. The liver was greatly enlarged, both in circumference and diameter, and weighed forty-two pounds. The kidneys were much enlarged and of a dark red color, due, no doubt, to blood extravasation. Their structure was also soft and easily broken down under pressure. The abdominal parietes were covered with a thick layer of adipose tissue. The heart was large, though not abnormally so; each ventricle was contracted and contained a small amount of dark, coagulated blood. The lungs were of a pale, anæmic color, due, no doubt, to the thorough draining out of the blood from their tissues. The bronchi were partly filled with frothy mucus. The spleen was much enlarged and engorged with blood of a dark brown color. The intestines were normal, with the exception of the mesentery and omentum, they being abnormally covered with fat. The flesh of the body was almost bloodless and of a firm consistence, so much so as to cause comment from a butcher who was standing by, he remarking that the flesh was as free from blood as that of any beef he had ever seen bled to death.

This horse had been lightly worked nearly every day for the past two years. During this time, although highly fed, he was never known to have been sick. The horse had not been subjected to any unusual exertion previous to the fatal attack—in fact, the horse at the time he was first noticed to be sick had been standing still for some time waiting for a load of goods. He could not have been kicked by his mate in the stable, as each horse stood in a separate stall or with several unused stalls between them. It would appear as if the liver, which had been gradually enlarging for a long time, had finally reached the limit of its distention and consolidation, and spontaneously its parenchymatous structure gave way under the great strain it had been so long subjected to. What seems most surprising in this case was the entire absence of all abdominal pain and sweating—two symptoms that have hitherto been considered classical by veterinary authors in rupture of the liver.

